# Transperineal pelvic drainage combined with lateral position to promote perineal wound healing after abdominoperineal resection: A prospective cohort trial

**DOI:** 10.1097/MD.0000000000029104

**Published:** 2022-04-08

**Authors:** An Shang, Min Wang, Yongping Yang, Zeyun Zhao, Donglin Li, Yu Guo, Rui Qi, Yang Yang, Shuang Wang

**Affiliations:** a Department of the General Surgery; b Department of Dermatology, The Second Hospital of Jilin University, Changchun, Jilin, China No. 218, Ziqiang Street, Nanguan District, Changchun City, Jilin Province, China.

**Keywords:** abdominoperineal resection, lateral position, perineal wound healing, perineal wound infection, transperineal wound drainage

## Abstract

**Background:**

For the rectal cancer <5 cm from anal margin, extralevator abdominoperineal resection (eAPR) has been accepted widely by surgeons. However, the rate of perineal infection following up eAPR is approximately 70%. We did the study with the aim of evaluating the effect and safety of transperineal pelvic drainage combined with lateral position (TPDLP) on perineal wound in patients undergoing eAPR.

**Methods:**

Patients were randomly assigned to N-TPDLP group (standard arm) or TPDLP group (intervention arm). In the standard arm, surgery was completed after abdominal drainage tube was placed in pelvic. Comparatively, an additional transperineal wound drainage tube was applied in the experimental arm. Postoperatively, patients of both 2 groups were informed not to sit to reduce perineal compression until the perineal wound healed. But lateral position was demanded in the intervention arm. The primary endpoint was the rate of uncomplicated perineal wound healing defined as a Southampton wound score of <2 at 30 days postoperatively. Patients were followed for 6 months.

**Results:**

In total, 60 patients were randomly assigned to standard arm (n = 31) and intervention arm (n = 29). The mean perineal wound healing time was 34.2 (standard deviation [SD] 10.9) days in TPDLP arm, which significantly differ from 56.4 (SD 34.1) in N-TPDLP arm (*P* = .001). At 30 days postoperatively, 3 (10%) of 29 patients undergoing TPDLP were classified into grade 4 according to Southampton wound score, however, 16 (52%) of 31 patients were classified into grade 4 in control arm, and significantly difference was observed between randomization groups (*P* = .001). What's more, perineal wound pain was assessed at 30 days postoperatively, and it is discovered that the pain degree of patients in control arm was significantly more severe than the interventive arm (*P* = .015).

**Conclusion:**

In the present study, we found that TPDLP generated a favorable prognosis for perineal wounds with acceptable side-effects.

## 1 Introduction

Colorectal cancer, the third most commonly diagnosed carcinoma and the third leading cause of carcinoma-related mortality, is still one of the mainly hazards for health of human.^[1]^ Currently, radical resection remains the standard treatment for early and even advanced colorectal cancer. For the rectal cancer <5 cm from anal margin, abdominoperineal resection (APR) had been accepted widely by surgeons since the first description for this surgical approach.^[2]^ However, traditional APR is associated with a high rate of positive margin due to its non-cylindrical resection without mesorectal removal at the level of the pelvic floor. Therefore, the traditional APR has been gradually abandoned in recent years.^[3]^ In 2007, extralevator abdominoperineal resection (eAPR) as an alternative procedure was first described by Holm et al,^[4]^ and then it was accepted by more and more surgeons. Several studies reported that eAPR performed better outcomes in circumferential resection margin positivity, rate of intraoperative perforation, and local recurrence compared with APR.^[5,6]^ However, after eAPR, a large cavity was created by resecting the anus, rectal, mesorectum, musculuslevatorani, and surrounding perineal skin. And then blood clot and exudate accumulate in the pelvic cavity, which increases the risk of pelvic abscess and perineal wound infection. Furthermore, the large cavity in perineal and the stiff structure of pelvic may increase the perineal wound tension followed by wound closure.^[7]^ Therefore, the rate of perineal infection in patients undergoing eAPR is approximately 70%.^[7–9]^

Perineal wound complications not only prolong the length of hospital stay, but also increase the cost of hospitalization. What's more, it may affect the daily life of patients undergoing APR and lead to a loss of quality of life (QOL).^[10,11]^ Several measures such as omentoplasty (OP), biological mesh closure of the pelvic floor, myocutaneous flaps, and incisional negative pressure wound therapy have been performed to reduce the probability of perineal wound complications for recent decades.^[9,12–14]^ However, OP was difficult to implement in some cases such as previous omental resection, metastatic involvement, and contracture owing to inflammation. Therefore, it is controversial whether OP is effective or not. Blok et al^[15]^ indicated that OP did not promote perineal wound healing; furthermore, it may result in additional morbidity and need for reintervention. Biological mesh closure of the pelvic floor was not superior in perineal wound healing and QOL in patients after eAPR and increased the duration of surgery, although it reduced the risk of perineal hernia compared with primary closure.^[9]^ For myocutaneous flaps, special techniques of plastic surgery and high risk of flap necrosis restricted its application.^[13]^ Nowadays, incisional negative pressure wound therapy was generally accepted by surgeons and it did decrease the risk of wound infection. However, for the patients with allergies, the new therapy seemed not available due to the use of film tape.^[14]^

Generally speaking, surgical wound infection was associated with microbiota diversity among the wound and microenvironment (dry, moist, and sebaceous). Microbes thrived in dark, moist, and nutrient-rich environments, which resulted in the wound infection eventually.^[16,17]^ In summary, wound adipose tissue liquefaction, remains of liquid beneath incision (such as effusion, residual blood clots, and ascites, etc) and sometimes high surface tension between sutured tissues could increase the risk of infection.^[18]^ Therefore, to apply transperineal pelvic drainage tube may be effective for perineal wound healing through keeping the wound dry. Furthermore, the application of lateral decubitus position could reduce the incision surface tension to some extent, and it could also reduce the stimulation of the perineal wound from pelvic fluid accumulation because the liquid will flow downwards.

Chen et al^[19]^ indicated that pelvic drainage tube combined with subcutaneous negative pressure drainage performed better efficacy and lower infection rate for perineal incision in laparoscopic-assisted abdominoperineal resection. Furthermore, Dinaux et al^[20]^ reported that prone position treatment for perineal wound during eAPR was associated with significantly lower perineal wound infection and dehiscence rates. However, there have been no studies reporting the effects of transperineal pelvic drainage combined with lateral position (TPDLP) for perineal wound in patients after eAPR. Therefore, further research is needed to evaluate whether TPDLP can promote the perineal wound healing in patients following the extralevator abdominoperineal resection.

We did the study with the aim of evaluating the effect and safety of TPDLP on perineal wound in patients undergoing eAPR.

## 2 Methods

### 2.1 Study design

The present research was a single center prospective clinical trial, performed in Second Affiliated Hospital of Jilin University. And the approval of the study protocol was obtained from the ethics committee of Second Affiliated Hospital of Jilin University. Eligible participators were randomized assigned to primary closure of perineal defect (standard arm) and transperineal pelvic drainage combined with lateral position after primary closure of perineal defect (intervention arm). An observer, unclear to the patient allocation, evaluated perineal wound healing using Southampton wound score after 30 and 60 days postoperatively. Sonography of perineal wound or pelvic computerized tomography (CT) was performed to evaluate the wound healing and perineal complications such as presacral sinus, perineal sinus, perineal abscess, and perineal herniation. In addition, during the follow-up, all severe events of perineal wound, containing medicine or surgical interventions and severe infections, were recorded.

### 2.2 Patients

A patient was considered eligible when the following conditions were satisfied: Age from 18 to 75 years; the distance from carcinoma to anal verge was <5 cm according to preoperative examination such as magnetic resonance imaging (MRI), CT, or colonoscopy; pathologically confirmed carcinoma; no evidences of distance metastasis were found after CT scan or MRI examination; able to complete postoperative follow-up. And the exclusion criteria were emergency surgery, synchronous primary tumors in colorectal or other organs, severe respiratory tract, liver, kidney, or cardiovascular disease, accepting neoadjuvant radiotherapy,^[21]^ and patients who enrolment in other trials that may affect the wound healing. After that, patients were randomized to TPDLP group and standard group. And the clinical date of patients was collected pre- and postoperative in the present research, which included baseline characteristics (such as age, sex, body mass index [BMI], previous surgery, comorbidity, tumor location, high-risk of invitation, preoperative radiotherapy, carcinoma embryonic antigen level, albumin levels 48 hours after surgery, and TNM stage according to postoperative pathology), surgical date (such as American Society of Anesthesiologists [ASA]-classification, operation time and bleeding volume during operation), and postoperative perineal wound healing date.

### 2.3 Randomization and masking

Stratified randomization was performed in the present study, and the stratified factors were age (18–59 or 60 years or older), sex (male or female), and surgical approach (open or laparoscopic surgery). After written informed consent, patients were assigned to 8 subgroups according to the stratified factors. After that, patients from each subgroup were randomly assigned to standard arm and intervention arm. The allocation of treatment was blinded to perineal wound assessor and patients.

### 2.4 Procedure

All patients were given antibiotics 2 hours before surgery according to the local trial site protocol. The operation method (such as invasive surgery or laparoscopic surgery) was left up to surgeons. The techniques of Biological mesh, omentoplasty and myocutaneous flaps had not been applied in our institution, therefore none of the patients had a mesh, omentoplasty or myocutaneous flap placed. In all patients, the principle of extralevator APR approach was adhered to, in which levator muscles were laterally resected, distal rectum and anal tube were excised completely. The coccyx was not routinely resected with the exception of surgical or carcinoma reasons. Finally, perineal defect was closed layer by layer with absorbable suture material.

In the standard arm, surgery was completed after placing abdominal drainage tube. Comparatively, an additional transperineal wound drainage tube was placed in the pelvic floor in the experimental arm. Postoperatively, patients of both 2 groups were informed not to sit to reduce perineal compression until the perineal wound healed. But lateral position was demanded in the intervention arm. Drainage tube was irrigated with 100 mL 0.9% saline solution everyday postoperatively to make sure the drainage tube is unobstructed in both 2 arms. Furthermore, antibiotics were routinely applied to patients from both 2 groups postoperatively.

### 2.5 Outcome

In the present study, the primary endpoint was the percentage of uncomplicated perineal wound healing which was defined as a Southampton wound score^[22]^ of <2 at 30 days postoperatively. The Southampton wound score is shown in Table 1. The second endpoint was wound infection rate 30 and 60 days after radical surgery, postoperative pain according to Visual Analogue Scale/Score and the time when perineal wound heal according to Southampton score. And the pain was defined as >3 on the scale for the present study, considered to potentially affect emotional or physical functioning.^[23]^ Other endpoints were perineal abscess rate, symptomatic and asymptomatic perineal hernia, perineal sinus rate, and other perineal complications requiring surgical intervention. The complications mentioned above were diagnosed through clinical signs and symptoms, clinical physical examination, laboratory tests, ultrasonic examination, and/or pelvic MRI scan. And the postoperative pain was defined as >3 on the scale in the present study, considered to potentially affect emotional or physical functioning.^[23]^

**Table 1:** Southampton wound score.

Grade	Appearance
0	Normal healing
I Normal healing with mild bruising or erythema:	
a	Some bruising
b	Considerable bruising
c	Mild erythema
II Erythema plus other signs of inflammation:	
a	At one point
b	Around sutures
c	Along wound
d	Among wound
III Clear or heamoserous discharge:	
a	At one point only (≤2 cm)
b	Along wound (>2 cm)
c	Large volume
d	Prolonged (>3 days)
IV Pus:	
a	At one point only (≤2 cm)
b	Along wound (>2 cm)
V Deep or severe wound infection with or without tissue breakdown; heamatoma requiring aspiration	

### 2.6 Statistical analyses

Statistical analyses were performed using SPSS for MAC, version 26.0 (IBM Corporation, Almonck, New York). Mann–Whitney *U* test or *t* test was used for continuous variables (e.g., age, BMI, operation time, bleeding volume during operation, perineal wound healing time, and length of stay). Chi-square test or Fisher exact test was used for comparing categorical data (e.g., gender, ASA-classification, previous surgery, comorbidity, tumor location, preoperative radiotherapy, high risk of invasion, TNM stage, normal perineal wound healing, infection rate, postoperative pain, perineal abscess rate, perineal hernia rate, persistent perineal sinus rate, and surgical reintervention rate). Multivariate analyses were evaluated with Cox proportional hazards models. The Kaplan–Meier curve was used to assess the perineal wound healing rate, and study arms were compared using a log rank test. And *P* < .05 was considered statistically significant.

## 3 Result

### 3.1 Recruitment

**Figure 1:**
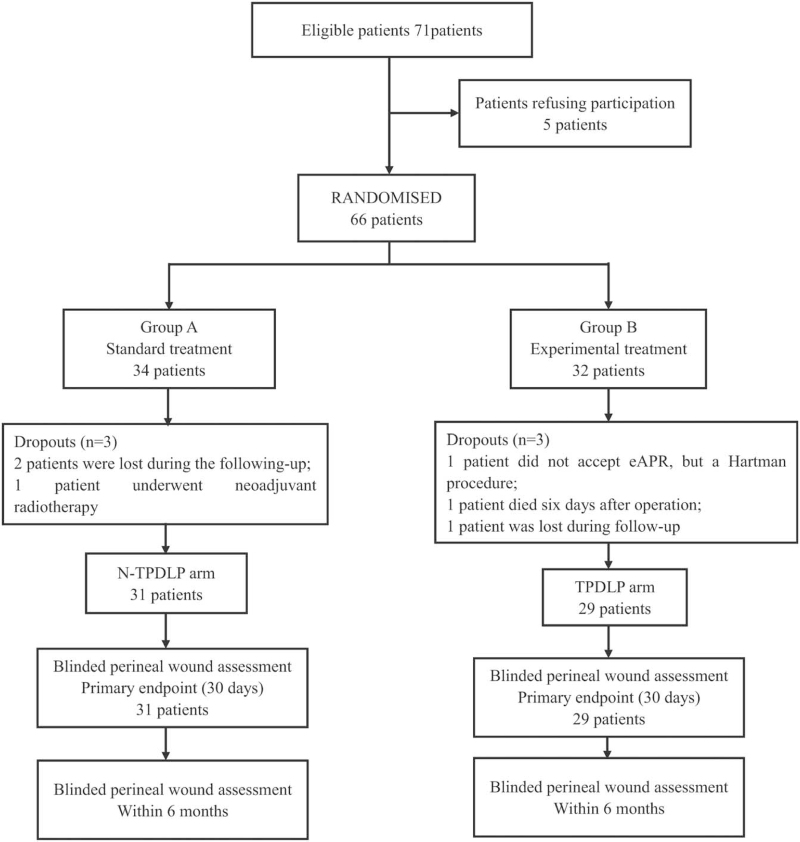
Flow diagram of the present prospective clinical trial.

Between the first of January 2018 and the first March of 2021, 71 eligible patients were approached to participate in the present study. Of the 71 eligible patients, 65 patients consented to the trail, and of which 32 patients were randomly assigned to TPDLP group (experimental arm) and others to N-TPDLP group (standard arm). After randomization, 1 patient did not accept eAPR, but a Hartman procedure; 1 patient died 6 days after operation and 1 patient was lost during follow-up in the intervention group. In the control group, 2 patients were lost during follow-up and 1 patient underwent neoadjuvant radiotherapy. These 6 patients were excluded owing to the fact that these patients could not be evaluated for the primary endpoint, resulting in 29 patients in the control group and 31 patients in the standard group (Fig. 1).

### 3.2 Baseline characteristics

The baseline characteristics of the included patients were described in Table 2. The mean age was 59.4 years (standard deviation [SD] 10.4) in N-TPDLP group and 60.5 years (SD 8.6) in TPDLP group (*P* = .659), and 60% (36/60) of the patients were men. The mean BMI was 23.4 kg/m^2^ (SD 3.4) in patients of intervention arm, which did not significantly differ from 23.8 kg/m^2^ (SD 3.8) in the control arm (*P* = .664). ASA-classification was performed in all 60 patients. 5 (16%) of the 31 patients were classified into grade 3, which did not significantly differ from 5 (17%) of the 29 patients in the TPDLP group (*P* = .908). The mean operation time was 195 minutes (SD 21) and 190 minutes (SD 12) separately in control group and experimental group, and no significant difference was observed (*P* = .273). And there was also no significant difference between N-TPDLP arm and TPDLP arm in bleeding volume during operation (77 mL, SD 22 vs 79 mL, SD 20; *P* = .665).

**Table 2:** Baseline characteristics of the 61 patients in the present study.

Baseline characteristics		Group A N-TPDLP (N = 31)	Group B TPDLP (N = 29)	*P*
Sex	Male (n, %)	16 (52)	20 (69)	.170
	Female (n, %)	15 (48)	9 (31)	
Age	years ± SD	59.4 (10.4)	60.5 (8.6)	.659
Body mass index	kg/m^2^ ± SD	23.8 (3.8)	23.4 (3.4)	.664
ASA-classification	ASA-2 (n, %)	26 (84)	24 (83)	.908
	ASA-3 (n, %)	5 (16)	5 (17)	
Previous surgery	Abdominal surgery (n, %)	5 (16)	4 (14)	.800
Comorbidity	Diabetes (n, %)	5 (16)	7 (24)	.438
	Hypertension (n, %)	6 (19)	6 (21)	.897
	Cardiac (n, %)	5 (16)	1 (3)	.102
	Vascular (n, %)	1 (3)	0 (0)	.329
	Smoking (n, %)	7 (23)	8 (28)	.655
Tumor location	<3 cm from lower border tumor to anal verge in MRI	14 (45)	10 (34)	.399
Laboratory examination	Elevated CEA level (n, %)	9 (29)	7 (24)	.668
	Hypoalbuminemia 48 h after APR (<3.0 g/dL) (n, %)	7 (23)	7 (24)	.887
High-risk of invasion	MRF (+) (n, %)	3 (10)	5 (17)	.389
	EMVI (+) (n, %)	6 (19)	6 (21)	.897
Operation time	minutes ± SD	195 (21)	190 (12)	.273
Bleeding volume during operation	mL ± SD	77 (22)	79 (20)	.665
TNM stage	Stage 1 (n, %)	8 (26)	7 (24)	.878
	Stage 2 (n, %)	10 (32)	8 (28)	
	Stage 3 (n, %)	13 (42)	14 (48)	

ASA = American Society of Anesthesiologists, BMI = body mass index, CEA = carcinoembryonic antigen, EMVI = extramural vascular invasion, MRF = mesorectal fascia, SD = standard deviation, TPDLP = transperineal pelvic drainage combined with lateral position. Statistically significant: *P* < .05.

### 3.3 Primary outcome

The perineal wound healing was evaluated with Southampton wound score in the present research, and perineal wound uncomplicated healing was defined as a Southampton wound score <2 at 30 days postoperatively. And the percentage of patients with uncomplicated perineal wound healing was 31% (9/29) in TPDLP arm, which did not significantly differ from 29% (9/31) in N-TPDLP arm (*P* = .866) (Table 3).

**Table 3:** Perineal wound healing.

Perineal wound healing		Group A N-TPDLP (N = 31)	Group B TPDLP (N = 29)	*P*
Normal perineal wound healing (Southampton wound score <2)	30 days postoperative (n, %)	9 (29)	9 (31)	.866
Perineal wound healing time	Days ± SD	56.4 (34.1)	34.2 (10.9)	.001
Severity of infection (at 30 days)	Erythema and other signs of inflammation (n, %)	5 (16)	4 (14)	.001
	Clear or hemoserous discharge (n, %)	4 (13)	17 (58)	
	Pus discharge (n, %)	15 (48)	3 (10)	
	Deep or severe wound infection (n, %)	3 (10)	0 (0)	
	Erythema and other signs of inflammation (n, %)	11 (35)	0 (0)	<.001
	Clear or hemoserous discharge (n, %)	6 (19)	1 (3)	
Severity of infection (at 60 days)	Pus discharge (n, %)	2 (6)	0 (0)	
	Deep or severe wound infection (n, %)	0 (0)	0 (0)	
	>3 according to VAS score (n, %)	8 (26)	1 (3)	.015
	Perineal abscess (n, %)	4 (13)	1 (3)	.36
Postoperative pain (at 30 days)	Perineal hernia (n, %)	3 (10)	0 (0)	.24
Other complications within 6 months	Persistent perineal sinus (n, %)	3 (10)	1 (3)	.61
	Surgical reintervention (n, %)	1 (3)	1 (0)	1.00
	Days ± SD	22 (7.0)	20.1 (5.0)	.377
LOS				

LOS = length of stay, SD = standard deviation, TPDLP = transperineal pelvic drainage combined with lateral position. Statistically significant: *P* < .05.

### 3.4 Secondary outcome

During the complete follow-up, the mean perineal wound healing time was 34.2 (SD 10.9) days in TPDLP arm, which significantly differ from 56.4 (SD 34.1) in N-TPDLP arm (*P* = .001). Furthermore, severity of infection according to the Southampton wound score was assessed at 30 and 60 days postoperatively. At 30 days postoperatively, the infective degree of 3 (10%) patients undergoing TPDLP were classified into grade 4 according to Southampton wound score, however, 16 (52%) patients were classified into grade 4 in control arm, and significant difference was observed between randomization groups (*P* = .001). At 60 days postoperatively, 11 (35%) of 31 patients in the control arm was classified into grade 2 according to Southampton wound score in the control arm, which significantly differ from 0 to 29 patients in interventive arm (*P* < .001). What's more, perineal wound pain was assessed at 30 days postoperatively, and the pain degree of patients in control arm was significantly more severe than the interventive arm (*P* = .015). However, the mean length of stay was 22 (SD 7.0) in control arm and 20.1 (SD 5.0) in intervention arm (*P* = .377). And no significant difference was observed between randomization groups in perineal abscess rate within 6 months postoperatively (*P* = .36). There was also no significant difference between 2 arms in perineal hernia rate (*P* = .24), persistent perineal sinus rate (*P* = .61), and surgical reintervention rate (*P* = 1.00) within 6 months postoperatively (Table 3).

To determine whether TPDLP was independent factor associated with perineal wound outcomes, a univariate and multivariate analysis was performed using the Cox proportional hazard model (Table 4). The risk variables included age, gender, BMI, diabetics, smoking, prior abdominal surgery, tumor location, elevated carcinoma embryonic antigen level, ASA-classification, hypoalbuminemia, operation time, and bleeding volume during operation. These factors were generally considered to be associated with prognosis of perineal wound outcomes. In the univariate analysis, TPDLP (HR 3.33, 95% CI 1.80–6.13, *P* < .001) was significantly associated with a better perineal wound outcome compared with other factors. In the final multivariable Cox regression model, TPDLP (HR 4.42, 95% CI 2.11–9.26, *P* < .001) independent of other factors was associated with a favorable prognosis of perineal wound. However, carcinoma locating <3 cm from anal verge (HR 0.44, 95% CI 0.21–0.95, *P* = .036) and men (HR 0.47, 95% CI 0.23–0.94, *P* = .034) independent of other factors was associated with a worse prognosis of perineal wound.

**Table 4:** Multivariable analysis of factors associated with time to perineal wound healing.

Characteristic	Univariate analysis		Multivariate analysis	
	HR (95% CI)	*P* value	HR (95% CI)	*P* value
TPDLP				
Did not receive	1.0 (Reference)		1.0 (Reference)	
Received	3.33 (1.80–6.13)	<.001	4.42 (2.11–9.26)	<.001
Age				
<65 yr	1.0 (Reference)		1.0 (Reference)	
≥65 yr	1.24 (0.73–2.09)	.425	1.14 (0.65–2.00)	.649
Gender				
Female	1.0 (Reference)		1.0 (Reference)	
Male	0.95 (0.56–1.62)	.862	0.47 (0.23–0.94)	.034
BMI				
<25 kg/m^2^	1.0 (Reference)		1.0 (Reference)	
≥25 kg/m^2^	1.13 (0.67–1.92)	.664	1.18 (0.64–2.15)	.598
Diabetics				
No	1.0 (Reference)		1.0 (Reference)	
Yes	1.59 (0.85–2.97)	.111	1.30 (0.58–2.95)	.523
Smoking				
No	1.0 (Reference)		1.0 (Reference)	
Yes	1.28 (0.71–2.30)	.391	1.47 (0.71–3.05)	.300
Prior abdominal surgery				
No	1.0 (Reference)		1.0 (Reference)	
Yes	1.79 (0.87–3.69)	.125	1.67 (0.74–3.74)	.215
Tumor location				
≥3 cm from anal verge	1.0 (Reference)		1.0 (Reference)	
<3 cm from anal verge	0.59 (0.34–1.01)	.052	0.44 (0.21–0.95)	.036
Elevated CEA level				
No	1.0 (Reference)		1.0 (Reference)	
Yes	0.92 (0.52–1.63)	.758	0.66 (0.33–1.31)	.237
ASA-classification				
II	1.0 (Reference)		1.0 (Reference)	
III	1.14 (0.57–2.28)	.704	0.80 (0.34–1.85)	.592
Hypoalbuminemia				
No	1.0 (Reference)		1.0 (Reference)	
Yes	0.73 (0.40–1.37)	.328	0.66 (0.33–1.31)	.230
Operation time				
<180 min	1.0 (Reference)		1.0 (Reference)	
≥180 min	0.92 (0.46–1.84)	.838	0.99 (0.41–2.37)	.977
Bleeding volume during operation				
<100 mL	1.0 (Reference)		1.0 (Reference)	
≥100 mL	0.94 (0.51–1.75)	.843	0.92 (0.39–2.15)	.847

ASA = American Society of Anesthesiologists, BMI = body mass index, CEA = carcinoembryonic antigen, TPDLP = transperineal pelvic drainage combined with lateral position. Statistically significant: *P* < .05.

Furthermore, Kaplan–Meier analyses were performed to further analyze the association of TPDLP and prognosis of perineal wound in patients after eAPR.

**Figure 2:**
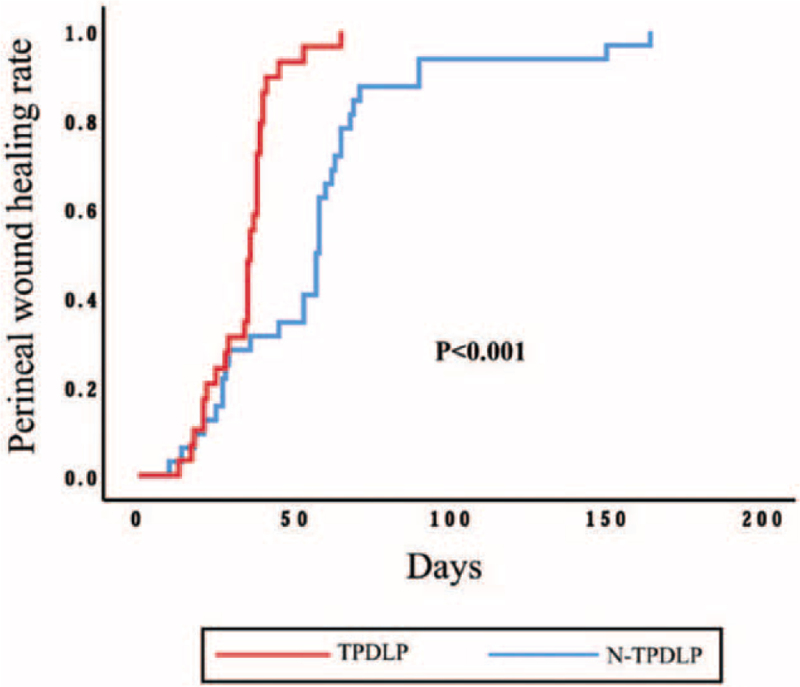
Kaplan–Meier curve of the perineal wound healing time.

At 30 and 60 days postoperatively, the perineal wound healing rates of the patients were 29% and 64% respectively in the standard arm, 31% and 97% respectively in the TPDLP arm (*P* < .001, Kaplan–Meier log-rank) (Fig. 2).

## 4 Discussion

Abdominoperineal resection (APR), as a radical surgery of rectal carcinoma <5 cm from anal verge, is the most widely accepted operation by surgeons.^[24]^ However, the high morbidity of perineal complications after APR has troubled medical profession for many years, which not only increases the hospitalization cost, but also reduces the QOL of the patients.^[11]^ Many elements (such as large wound tension, hematocele, or effusion around the wound and propagation of microorganisms) were considered to be associated with perineal complications.^[16]^ Therefore, if TPDLP was put into effect, the risk factors of perineal complications would be eliminated partly, and the prognosis of perineal wound would be better.

The present study elicited 3 main findings. First, although no significant difference was observed in uncomplicated perineal wound healing between the 2 randomization groups (31% in TPDLP arm vs 29% in N-TPDLP arm, *P* = .866), the mean perineal wound healing time (34.2 SD 10.9) in TPDLP arm was significantly shorter compared with the patients in control arm (56.4 SD 34.1) (*P* = .001). Second, the perineal wound infection degree of the patients in intervention arm was lower compared with patients in control group at both 30 and 60 days postoperatively. Finally, the pain degree of the patients in control group was more severe than patients undergoing DPDLP at 30 days postoperatively.

Several randomized prospective studies comparing different interventions such as omentoplasty (OP), biological mesh closure of the pelvic floor, myocutaneous flaps, and incisional negative pressure as a strategy to promote perineal wound healing have been published over recent decades. However, studies related to transperineal pelvic drainage combined with lateral position for perineal wound treatment were quite rare. The retrospective literature of Abdominoperineal Resection for Rectal Cancer: Is the Pelvic Drain Externalization Site an Independent Risk Factor for Perineal Wound Healing^[25]^ indicated that patients treated for transperineal drainage tube postoperatively showed better wound outcomes with statistically significant lower delayed wound healing rate. The result was congruent with the result in the present study when only considering the difference of perineal wound healing time after eAPR between the 2 groups. However, no significant differences in perineal wound healing rates at 30 days postoperatively were witnessed between TPDLP group and control group (31% vs 29%, *P* = .866) in the present study, which was not consistent with the viewpoint of Pramateftakis et al.^[25]^ The distinction may be related to the following 2 elements. First, drainage tube as a foreign matter for body may induce aseptic inflammation around the tube, and the wound healing near the drainage tube would be delayed by 1 to 2 weeks after the tube pulled out. Second, the number of eligible patients was small, and the difference may be statistically significant if more patients enrolled in the present research.

In the present study, multivariate analysis was performed to further evaluate the independent factors associated with perineal wound outcomes. TPDLP was associated with a favor perineal wound prognosis for patients after eAPR (HR 4.42, 95% CI 2.11–9.26, *P* < .001), however, men (HR 0.47, 95% CI 0.23–0.94, *P* = .034) and carcinoma locating <3 cm (HR 0.44, 95% CI 0.21–0.95, *P* = .036) from anal verge were associated with a poor outcome. The literature of predictors of wound dehiscence and its impact on mortality after abdominoperineal resection: data from the National Surgical Quality Improvement Program^[26]^ put forward that men independent of other factors was associated with a worse prognosis of perineal wound (HR 2.032, 95% CI 1.126–3.666, *P* = .019), which was concurrent with the present study. On the contrary, the research of Predictors of Perineal Wound Complications and Prolonged Time to Perineal Wound Healing After Abdominoperineal Resection^[27]^ indicated that hypoalbuminemia was associated with poor outcomes of perineal wound (HR 11.37, 95% CI 2.39–54.03, *P* = .002), which was not consistent with the present study. The different result may be attributed to the little enrolled patients in the present study. Hypoalbuminemia was also associated with a worse prognosis of perineal wound in this research, and the risk factor may be statistically significant if more eligible patients were enrolled. Furthermore, the present prospective cohort trial indicated that patients treated with TPDLP performed a lower severity of perineal wound infection according to Southampton wound score, which was consistent with the viewpoint of Zeng et al.^[28]^

However, Nakayama et al^[29]^ indicated that the aplication of drainage did not prevent the wound infections in patients undergong abdominal surgery. The following 2 reasons may account for the difference between the 2 literature. First, the drainage was used for abdominal wound in Nakayama's study while TPDLP was applied into perineal wound in the present trial. Perineal wound, in general, was more easily influenced by abdominal or pelvic hydrops due to its lower position compared with abdominal wound. Therefore, perineal wound drainage tube may be more significative than abdominal wound. Second, restricting patients to lateral position postoperatively may prevent the perineal wound infection through reducing the wound stimulation in the present trial.

Transperineal wound drainage tube combined with lateral position was performed in patients after eAPR in the present research. Previous clinical trial had reported that non-drainage was associated with higher risk of deep infection in patients after total hip arthroplasty.^[28]^ Therefore, the drainage application for perineal wound may also possess a favor wound prognosis because the 2 wounds were similar in high tension as the suture. More importantly, surgical wound infection was associated with microbiota diversity among the wound and microenvironment (dry, moist, and sebaceous),^[17]^ and the moist environment (such as hematocele and hydrops) where bacteria thrives may be eliminated through transperineal wound drainage, which provided theoretical basis for the present trial. In addition, transperineal wound drainage was more adequate compared with transabdominal wound drainage, after all, the perineum is the lowest point of the pelvic cavity for human with erect position. Wiatrek et al^[7]^ indicated that the perineal wound tension was related to the incidence of complications of perineal wounds, and lateral decubitus position should be used to reduce wound tension, which could also reduce the stimulation of the perineal wound from pelvic fluid accumulation because the liquid will flow downwards.

The present study had several limitations. First, this was a single central prospective clinical trial, and a multi-central study would be idealized. Second, a sample size of the present clinical trial was still small because the patients suffering lower rectal carcinoma were few during the past 3 years in Second Affiliated Hospital of Jilin University. But a sample size of 61 patients was also acceptable for prospective studies. Finally, it was challenging for evaluators to objectively assess the perineal wounds, especially when wound scoring systems were limited and not validated for every type of wounds. Therefore, the Southampton wound score was the best available method for this purpose. At last, a prospective and multi-center study with a large sample size is required to further evaluate the efficiency and safety of TPDLP in the future.

## 5 Conclusion

In the present study, we found that TPDLP generated a favorable prognosis for perineal wounds with acceptable side-effects. Thus, TPDLP may be a promising and exciting therapeutic strategy for patients suffering abdominal perineal resection.

## Acknowledgments

Jian Shi, Colorectal Section, Department of Surgery, The Second Hospital of Jilin University, No. 218, Ziqiang Dist, Changchun, 130041, Jilin.

## Author contributions

AS and SW conceived the study design. MW and YY acquired the data for the study. ZZ, DL, and YG analyzed and interpreted the data. AS drafted the manuscript. MW and YY revised the manuscript critically. The authors read and approved the final manuscript.

**Data curation:** Zeyun Zhao, Yu Guo, Rui Qi, Yang Yang.

**Formal analysis:** Donglin Li.

**Methodology:** Yongping Yang.

**Writing – original draft:** An Shang.

**Writing – review & editing:** Shuang Wang, Min Wang.
